# Network Pharmacology and In Vivo Experimental Validation to Uncover the Renoprotective Mechanisms of Fangji Huangqi Decoction on Nephrotic Syndrome

**DOI:** 10.1155/2022/4223729

**Published:** 2022-06-08

**Authors:** Jiazhen Yin, Dongrong Yu, Lichan Mao, Caifeng Zhu, Jin Yu

**Affiliations:** Department of Nephrology, Hangzhou TCM Hospital Affiliated to Zhejiang Chinese Medical University (Hangzhou Hospital of Traditional Chinese Medicine), Hangzhou 310007, China

## Abstract

**Background:**

Fangji Huangqi decoction (FHD) is a traditional Chinese medicine formula that has the potential efficacy for nephrotic syndrome (NS) treatment. This study aims to explore the effects and underlying mechanisms of FHD against NS via network pharmacology and in vivo experiments.

**Methods:**

The bioactive compounds and targets of FHD were retrieved from the TCMSP database. NS-related targets were collected from GeneCards and DisGeNET databases. The compound-target and protein-protein interaction networks were constructed by Cytoscape 3.8 and BisoGenet, respectively. GO and KEGG analyses were performed by the DAVID online tool. The interactions between active compounds and hub genes were revealed by molecular docking. An NS rat model was established to validate the renoprotective effects and molecular mechanisms of FHD against NS in vivo.

**Results:**

A total of 32 hub genes were predicted to play essential roles in FHD treating NS. Eight main bioactive compounds of FHD had the good affinity with 9 hub targets (CCL2, IL-10, PTGS2, TNF, MAPK1, IL-6, CXCL8, TP53, and VEGFA). The therapeutic effect of FHD on NS was closely involved in the regulation of inflammation and PI3K-Akt pathway. In vivo experiments confirmed the renoprotective effect of FHD on NS, evidenced by reducing the levels of proteinuria, serum creatinine, blood urea nitrogen, and inflammatory factors in NS rats. The PI3K activator 740Y-P weakened the effects of FHD against NS. Furthermore, FHD downregulated the levels of PTGS2, MAPK1, IL-6, and p-Akt in NS rats.

**Conclusions:**

FHD alleviates kidney injury and inflammation in NS by targeting PTGS2, MAPK1, IL-6, and PI3K-Akt pathway.

## 1. Introduction

Nephrotic syndrome (NS) is a clinical syndrome commonly characterized by edema, hyperlipidemia, hypoalbuminemia, and heavy proteinuria [1]. The incidence of NS is caused by diverse reasons, including auto-antibodies to specific glomerular antigens, genetic mutations, infections, metabolic disorders, podocytopathies, or paraneoplastic syndromes [2]. NS is one of the most common types of chronic kidney disease diagnosed in clinic. It has been reported that the incidence of NS accounts for 40% of all the renal biopsies, in which primary NS accounts for 75% [3]. At present, the pathogenesis of NS is not clear, and the therapeutic approaches are limited. As a consequence, there is an urgent need for a safer and more effective drug against NS.

Traditional Chinese medicines (TCMs) have attracted great attention for NS treatment, due to their favorable properties of low side effects, recurring rate, and cost [4]. Importantly, TCMs exert therapeutic effects by coordinating multicompounds and multitargets. Fangji Huangqi decoction (FHD), one of the classical TCMs formula, is composed of four medicinal herbs including Stephania Tetrandra Radix (Fangji, FJ), Astragali Radix (Huangqi, HQ), *Glycyrrhiza* Radix (Gancao, GC), and Atractylodis Macrocephalae Rhizoma (Baizhu, BZ). As the main herb in the FHD, FJ has been reported to possess various pharmacological properties, such as immunomodulatory, anti-inflammatory, anticancer, antifibrotic, antiplatelet, and antidiabetic effects [5]. Similarly, HQ, GC, and BZ also have been demonstrated to exhibit commendable pharmacological activities, including immunomodulation, antioxidation, inhibition of liver fibrosis, cardiovascular protection, stimulation of blood regeneration, and pain-relieving properties [6–8]. However, potential mechanisms of FHD in the treatment of NS are still illusive.

TCMs are characterized by multicomponent and multitarget, which exert therapeutic effects on diverse diseases via bioactive components acting on multiple targets [9]. Network pharmacology, an emerging and promising subject, is a powerful tool to explore the associations between active components and potential targets for TCMs treating complex diseases [10]. Network pharmacology systematically studies the interaction between “drug-target-pathway-disease” based on the integration of bioinformatics, molecular biology, and public database information. Varghese and Majumdar proposed network pharmacology as an initial inherent approach in identifying the repurposing and synergism of drug candidates for NS treatment [11]. Some previous studies have identified the efficacy and underlying mechanisms of TCMs treating NS via network pharmacology [12, 13]. For instance, Yiqi Huoxue Decoction has been found to enhance renal function and alleviate podocyte injury in NS, and the potential mechanisms involved PI3K-AKT and NF-*κ*B pathways based on network pharmacology [12]. Duan et al. established the underlying mechanism of Qingrekasen granule against NS, including promoting autophagy and antiapoptosis through regulating the expression of AKT1, CASP3, BCL2L1, and mTOR to protect podocytes and maintain renal tubular function [13]. Furthermore, Zhang et al. revealed that FHD possesses the antinephrotic syndrome effect in rat nephropathy model; however, the relative underlying mechanisms have not been fully elucidated [14]. In this study, a systematic network pharmacology approach was applied to predict the bioactive components and hub targets for FHD against NS. Moreover, in vivo experiments were performed to validate the therapeutic efficacy of FHD on NS, and the potential mechanism (PI3K-Akt pathway) and hub targets [prostaglandin-endoperoxide synthase 2 (PTGS2), mitogen-activated protein kinase 1 (MAPK1), and interleukin 6 (IL-6)] were uncovered. Also, a total of 144 bioactive compounds of FHD and 62 overlapping targets of FHD against NS were discovered for further investigation. This study provides a potential therapeutic drug for NS and offers the essential foundation for the mechanism investigation of FHD treating NS.

## 2. Materials and Methods

### 2.1. Screening of Bioactive Compounds and Targets of FHD

The active compounds and targets of FHD were retrieved from the TCM systems pharmacology (TCMSP) analysis platform (https://tcmspw.com/tcmsp.php). All potential active compounds were subjected to absorption, distribution, metabolism, and excretion (ADME) of drugs screened with the criteria of oral bioavailability (OB) ≥ 30% and drug-likeness (DL) ≥ 0.18 [15]. Besides, bioactive compounds with targets >3 were also selected as candidates. More active components of FHD with significant pharmacological effects were supplemented based on previous literature. Subsequently, target proteins of active compounds were predicted in SwissTargetPrediction database (https://www.swisstargetprediction.ch/) with the criteria of probability >0.5.

### 2.2. Construction of Herb-Compound-Target (H-C-T) Network

Active compounds of FHD and their corresponding targets were subjected to establish the H-C-T network. H-C-T network was constructed by Cytoscape (version 3.8, https://www.cytoscape.org/), an open software package project for visualizing, integrating, modeling, and analyzing the interaction network [16].

### 2.3. Screening of NS-Related Targets

The therapeutic targets for NS were obtained from GeneCards database (https://www.genecards.org) [17] and DisGeNET database (https://www.disgenet.org/) [18]. The words “minimal change disease,” “membranous nephropathy,” “focal segmental glomerulosclerosis,” “mesangial proliferative glomerulonephritis,” and “mesangial capillary glomerulonephritis” were used as index words, and the species were limited to “*Homo sapiens*” in the collection of therapeutic targets. The targets belonging to both databases were retained as candidate targets. STRING database (https://string-db.org/) was applied to construct the NS targets network based on the candidate NS-related therapeutic targets [19].

### 2.4. Construction of Protein-Protein Interaction (PPI) Network

The overlapping targets of disease and drug component were mapped with Venn diagram by online tools (https://bioinformatics.psb.ugent.be/webtools/Venn/) for further analysis. The Cytoscape plugin BisoGenet was conducted for the construction of PPI network for overlapping targets. The PPI network was thereafter inputted into Cytoscape for visualization. The PPI network was analyzed by Cytoscape plugin CytoHubba, and the hub genes were screened with the criteria of degree more than twice the median.

### 2.5. Gene Ontology (GO) and Kyoto Encyclopedia of Genes and Genome (KEGG) Pathway Enrichment Analyses

The Database for Annotation, Visualization and Integrated Discovery (DAVID, https://david.ncifcrf.gov/) was used to perform GO and KEGG pathway enrichment analyses with *P* < 0.05. The enriched GO and KEGG pathway terms were visualized by the Matplotlib [20].

### 2.6. Molecular Docking

The 2D structure of the eight core compounds were downloaded from Zinc 15 [21]. The compound's structures were added into AutoDockTools (version 1.5.6) [22] to add polar hydrogen, distribute charge, set rotatable keys, and save as “PDBQT” file. The 3D structures of the top nine hub genes were downloaded from the Protein Data Bank (PDB) database [23]. Separation of the target proteins from the original ligand and removal of water molecules was performed using PyMOL (version 2.3.0) [24]. The ligand and receptor were imported into AutoDockTools (version 1.5.6) to add polar hydrogen, distribute charge, and save as “PDBQT” file. Molecular docking was performed using AutoDock Vina (version 1.1.2) [25], and interaction pattern analysis was carried out using PyMOL to map combinations with the lowest binding energy (affinity).

### 2.7. In Vivo Experimental Validation

#### 2.7.1. Experimental Animals and Drug Treatment

All animal experiments were approved by the Institutional Animal Care and Use Committee and the local experimental ethics committee. Male Sprague-Dawley (SD) rats (*n* = 36) were purchased from Shanghai SLAC Laboratory Animal Co., Ltd. (Shanghai, China). Rats were kept under a 12 h light/dark cycle with 45 ± 15% humidity at 23 ± 1°C and had free access to food and water. After one week, SD rats were randomly divided into six groups (*n* = 6 per group): the control group (no treatment); the model group; the low-FHD group; the mid-FHD group; the high-FHD group; and the FHD + 740Y-P group. In order to induce NS, rats in the model and drug-treated groups were intravenously injected with 4 mg/kg adriamycin (ADR) and then injected with 1 mg/kg ADR after one week. Rats in the control group were intravenously injected with saline. After modeling, NS rats in the low-, mid-, and high-FHD groups were, respectively, administrated with 1, 2, and 4 g/kg/d FHD by oral gavage for 30 days. NS rats in the FHD + 740Y-P group were gavaged with 4 g/kg/d FHD and intraperitoneally injected with 20 *μ*M 740Y-P (a PI3K activator). Rats in the control and model group received equal volumes of water. The body weights of rats were recorded once every 4 weeks. After treatment, 24 h urines of all rats were collected to measure the level of urine protein using a BCA kit (Thermo Fisher Scientific, IL, USA). At the end point of treatment, all rats were anesthetized with 10% sodium pentobarbital and blood samples were collected via retro-orbital puncture. Rats were sacrificed by cervical dislocation and right kidneys were removed and weighed to calculate the kidney index (kidney weight/body weight).

### 2.8. Measurement of Inflammatory Factors and Renal Function Indicators

Blood samples were collected from orbits of rats and the serum was separated via centrifuging for 20 min at 3,000 r/min. The levels of serum inflammatory factors (IL-1*β*, MCP-1, and TGF-*β*1) were measured by the corresponding enzyme-linked immune-sorbent assay (ELISA) kits (Mlbio, China). For the assessment of renal function, the levels of serum creatinine (SCr), blood urea nitrogen (BUN), and albumin (ALB) were determined using a chemical analyzer (Fujifilm, Japan).

### 2.9. Hematoxylin and Eosin (H&E) Staining

Right kidneys of rats were removed and fixed in 4% formaldehyde for 24 h. Next, kidney tissues were dehydrated in alcohol and then embedded in paraffin. Tissues were sliced into 4  *μ*m-thick sections and went through dewaxing and hydration. Followed by that, sections were stained with hematoxylin for 5 min and with eosin for 2 min. The renal pathological changes were observed under a light microscope (Olympus, Japan).

### 2.10. qRT-PCR

Total RNA of kidney tissues was extracted using TRIzol reagent (Invitrogen, CA, USA) and were reversely transcribed to cDNA using the PrimeScript One-Step RT-PCR Kit (Takara, Japan). qRT-PCR was performed using the SYBR Green qPCR master Mix kit and the Agilent Stratagene Mx3000P Real-Time PCR instrument (DBI Bioscience, China). Reaction program was 95°C for 3 min, and 40 cycles of 95°C for 12 s and 62°C for 40 s. The primers used in this work were listed in Table S1. The relative mRNA expression of target genes was calculated using the 2^−∆∆Ct^ method by normalizing to GAPDH.

### 2.11. Western Blot Analysis

Total protein was extracted from kidney tissues using the lysis buffer (Beyotime, China) and quantified using the BCA kit (Thermo Fisher Scientific, MA, USA). Proteins were separated on 10% SDS-PAGE and then transferred onto polyvinylidene fluoride membranes (Millipore, MA, USA). After blocking with 5% skim milk for 1 h, membranes were incubated with primary antibodies at 4°C overnight. Then, membranes were incubated with horseradish peroxidase (HRP)-conjugated secondary antibodies for 1 h under dark condition. Protein bands were visualized using the ECL Substrate Kit (Abcam, UK) and photographed under the ChemiDoc Imaging System (Bio-Rad, CA, USA). The primary antibodies used in Western blotting were rabbit monoclonal anti-PTGS2 (1 : 1,000; GST, MA, USA), mouse monoclonal anti-IL-6 (1 : 500; Abcam, UK), rabbit monoclonal anti-MAPK1 (1 : 2,000; GST), rabbit polyclonal anti-Akt (1 : 1,000; Abcam), rabbit polyclonal anti-p-Akt (1 : 500; Abcam), and mouse monoclonal anti-GAPDH (1 : 500; Abcam). The secondary antibodies were rabbit anti-mouse IgG H&L and goat anti-rabbit IgG H&L (1 : 2,000; Abcam).

### 2.12. Statistical Analysis

All data were processed on the GraphPad 7.0 and presented as mean ± standard deviation. The comparison between groups was analyzed using the one-way ANOVA, followed by Tukey's test. *P* < 0.05 was taken as the measure of statistical difference.

## 3. Results

### 3.1. H-C-T Network of FHD

**Figure 1:**
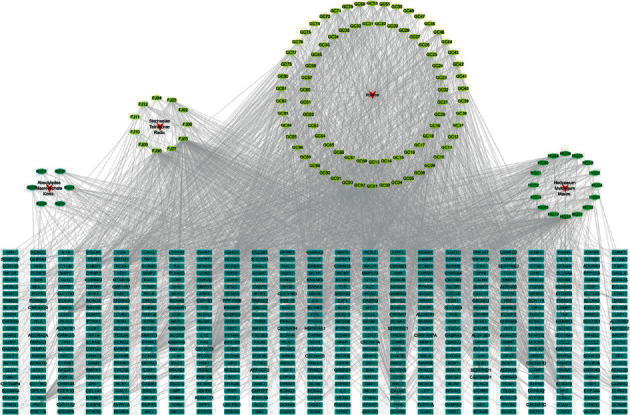
Herb-compound-target network of Fangji Huangqi decoction (FHD). Red arrows represent 4 herbs, green and yellow circles represent compounds, and blue rectangles represent targets.

According to the public database and previous literature, a total of 114 bioactive compounds of FHD were collected (Table S2). There were 11 active compounds from Stephaniae Tetrandrae Radix (FJ), 16 from Astragali Radix (HQ), 6 from Atractylodes macrocephalae Rhizoma (BZ), and 81 from Liquiritiae Radix et Rhizoma (GC). Subsequently, a total of 552 targets of these bioactive compounds were obtained from SwissTargetPrediction database. A H-C-T network was constructed to visualize the interactions between compounds and targets of FHD. This H-C-T network included 670 nodes (4 herbs, 114 compounds, and 552 targets) and 2781 edges (interactions between two target proteins) with an average degree of 8.418 (Figure 1).

### 3.2. Identification of Overlapping Targets for FHD Against NS

**Figure 2:**
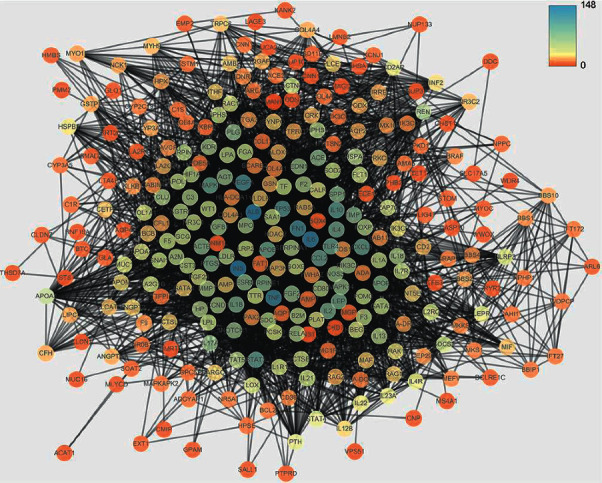
Protein-protein interaction (PPI) network of nephrotic syndrome- (NS-) related targets. Colors from orange to blue are proportional to the degrees of nodes.

**Figure 3:**
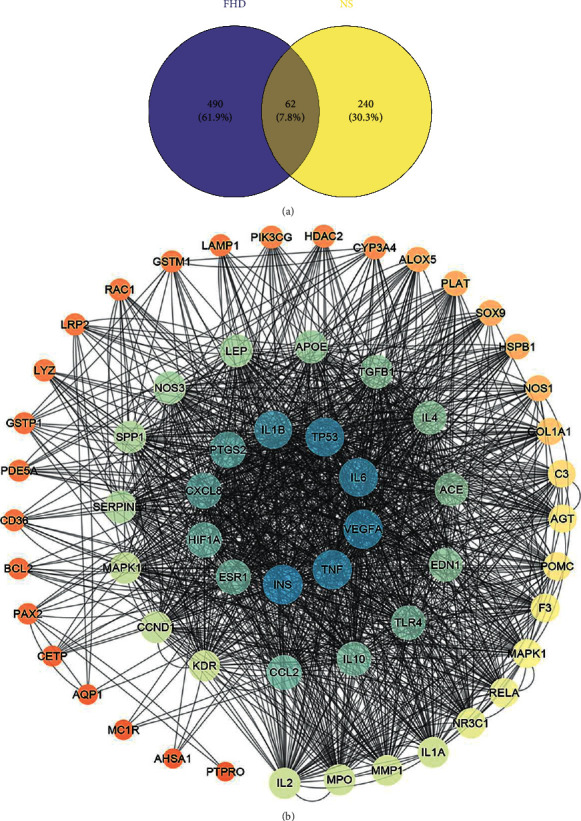
PPI network of potential therapeutic targets for FHD against NS. (a) Venn map of overlapping targets on FHD and NS. The purple circle indicates the targets of FHD, and the yellow circle indicates the targets of NS. The part of the two intersecting circles is the overlapping targets. (b) PPI network of overlapping targets on FHD and NS. Colors from orange to blue are proportional to the degrees of nodes.

Based on the public databases, 302 NS-related targets were obtained and a PPI network of these targets was constructed. The PPI network of NS-related targets included 297 nodes and 4361 edges with an average degree of 29.4 (Figure 2). In addition, a Venn diagram showed that there were 62 overlapping targets obtained by comparing the targets of FHD and NS, which were deemed to be potential therapeutic targets of FHD against NS (Figure 3(a)). The interactions among 62 overlapping targets were visualized by a PPI network. As shown in Figure 3(b), the PPI network of overlapping targets included 62 nodes and 749 edges with an average degree of 22.4. The top 25 hub targets were screened including INS, TNF, VEGFA, IL6, TP53, IL1B, PTGS2, CXCL8, HIF1A, ESR1, CCL2, IL10, TLR4, EDN1, ACE, IL4, TGFB1, APOE, LEP, NOS3, SPP1, SERPINE1, MAPK1, CCND1, and KDR (Figure 3(b)).

### 3.3. GO and KEGG Pathway Enrichment Analysis

**Figure 4:**
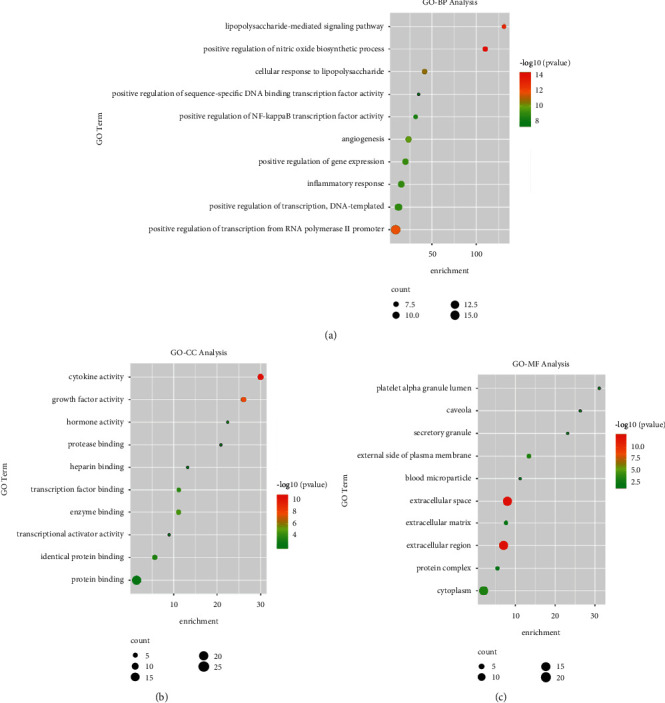
Gene ontology (GO) enrichment analysis for hub target genes of FHD treating NS. (a) The top 10 enriched terms in biological process (BP). (b) The top 10 enriched terms in cellular component (CC). (c) The top 10 enriched terms in molecular function (MF).

DAVID was applied for the GO enrichment and KEGG pathway enrichment of the 62 hub targets. The results of GO enrichment analysis indicated that 67 biological processes (BP), 4 cellular components (CC), and 10 molecular functions (MF) were obtained. In the BP ontology, the targets of the PPI network primarily associated with positive regulation of nitric oxide biosynthetic process, lipopolysaccharide-mediated signaling pathway, positive regulation of transcription from RNA polymerase II promoter, cellular response to lipopolysaccharide, angiogenesis, inflammatory response, positive regulation of NF-*κ*B transcription factor activity, and positive regulation of sequence-specific DNA binding transcription factor activity (Figure 4(a)). For the CC ontology, the targets located mainly in extracellular space, extracellular region, external side of plasma membrane, platelet alpha granule lumen, caveola, secretory granule, extracellular matrix, blood microparticle, and protein complex (Figure 4(b)). Based on GO annotation of MF, it could be seen that the targets were mainly involved in cytokine activity, growth factor activity, enzyme binding, transcription factor binding, hormone activity, heparin binding, and transcriptional activator activity (Figure 4(c)).

**Figure 5:**
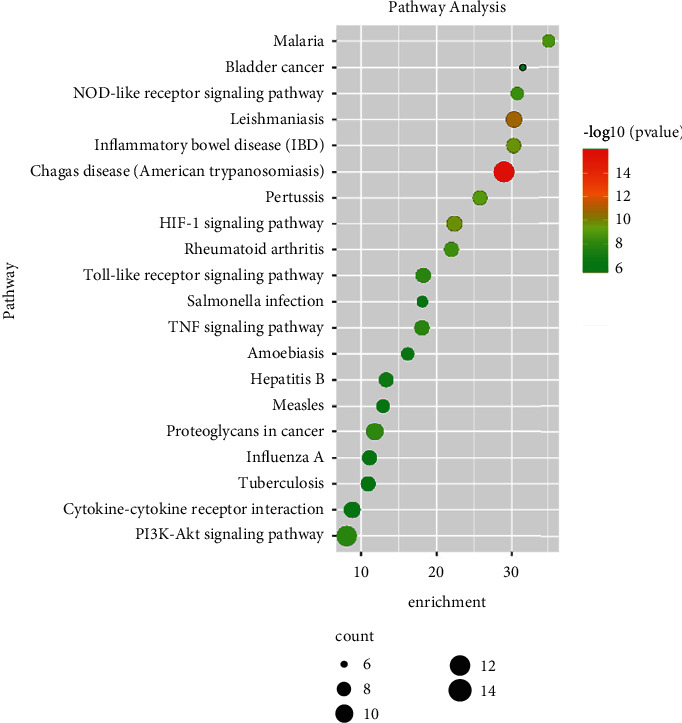
Kyoto Encyclopedia of Genes and Genomes (KEGG) pathway analysis for hub target genes of FHD treating NS. The color of circles represents the *P* value, and the size of circles represents the count.

The results of KEGG pathway enrichment revealed that the hub targets mainly related to HIF-1 signaling pathway, NOD-like receptor signaling pathway, proteoglycans in cancer, PI3K-Akt signaling pathway, Toll-like receptor signaling pathway, and TNF signaling pathway (Figure 5). The top 30 GO terms and top 20 KEGG pathways of hub targets were, respectively, shown in Tables S3 and S4.

### 3.4. Molecular Docking of Core Compounds and Hub Genes

**Figure 6:**
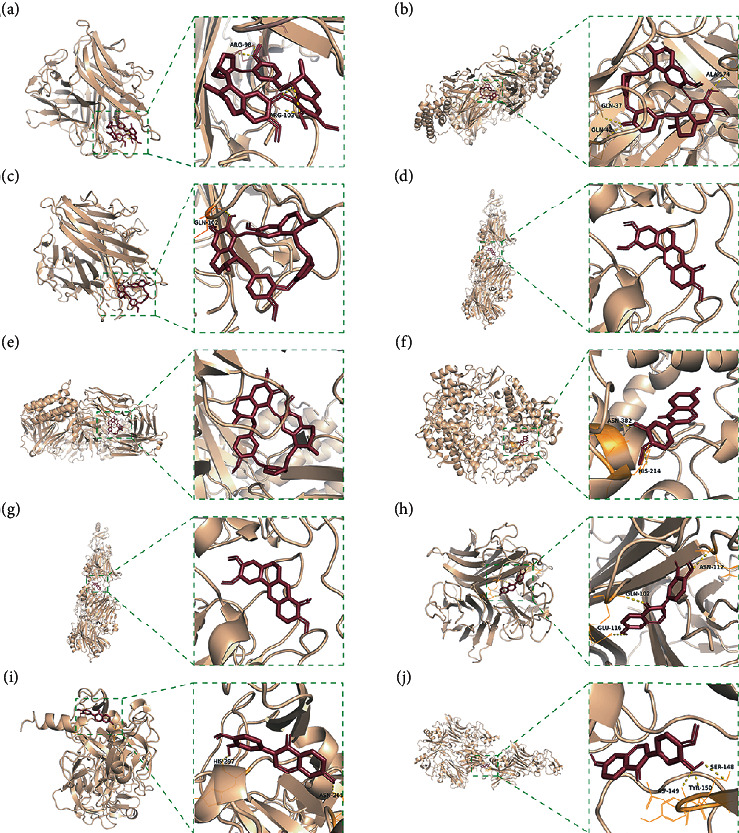
Molecular docking of bioactive compounds and hub targets for FHD treating NS. (a) TNF and tetrandrine, affinity = −10.1 kcal/mol. (b) IL10 and fangchinoline, affinity = −9.5 kcal/mol. (c) TNF and fangchinoline, affinity = −9.5 kcal/mol. (d) TNF and palmatine, affinity = −8.6 kcal/mol. (e) IL10 and tetrandrine, affinity = −8.6 kcal/mol. (f) PTGS2 and calycosin, affinity = −8.6 kcal/mol. (g) CCL2 and palmatine, affinity = −8.5 kcal/mol. (h) TNF and calycosin, affinity = −8.5 kcal/mol. (i) TP53 and calycosin, affinity = −8.3 kcal/mol. (j) CCL2 and calycosin, affinity = −8.2 kcal/mol.

The binding affinity between core compounds and hub genes of FHD were verified by molecular docking. As shown in Table 1, the eight bioactive compounds were palmatine, *β*-elemene, tetrandrine, fangchinoline, bifendate, calycosin, atractylenolide I, hinesol. The 9 hub targets were CCL2, IL10, PTGS2, TNF, MAPK1, IL6, CXCL8, TP53, and VEGFA. AutoDock Vina assessed the binding strength of small molecules and proteins primarily through affinity. A threshold affinity < −5 kcal/mol was set in this study. A total of 58 docking pairs were obtained as shown in Table S5. Tetrandrine showed the high binding affinity of −10.1 kcal/mol with TNF and −8.6 kcal/mol with IL10. Fangchinoline showed the high binding affinity of −9.5 kcal/mol with IL-10 and −9.5 kcal/mol with TNF. Palmatine showed the high binding affinity of −8.6 kcal/mol with TNF and −8.5 kcal/mol with CCL2. Calycosin showed the high binding affinity of −8.6 kcal/mol with PTGS2, −8.5 kcal/mol with TNF, −8.3 kcal/mol with TP53, and −8.2 kcal/mol with CCL2. The small-molecule compounds were tightly bound to the protein residues via various interactions (Figure 6).

### 3.5. In Vivo Experimental Validation

#### 3.5.1. Effects of FHD on Body Weight, Kidney Weight, Proteinuria, and Inflammatory Response in NS Rats via Affecting PI3K-Akt Signaling Pathway

**Figure 7:**
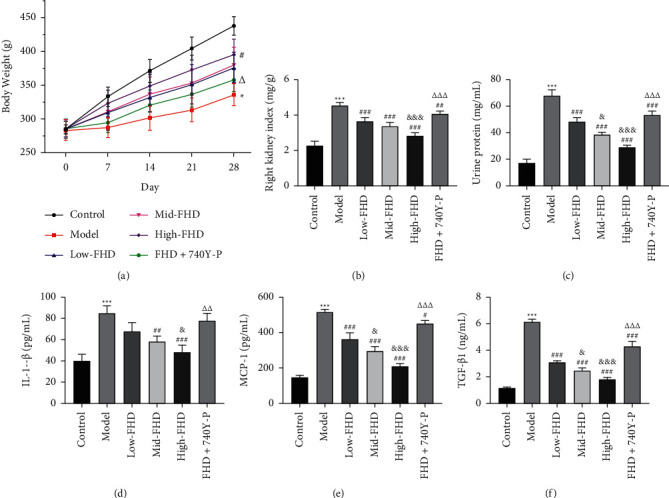
Effects of FHD on body weight, kidney weight, proteinuria, and inflammatory response in NS rats via affecting PI3K-Akt signaling pathway. (a) The body weight of rats was recorded once every 4 weeks after treatment. (b) The right kidney index (kidney weight/body weight) of rats. (c) The level of 24 h urine protein in rats was measured using a BCA kit. (d-f) The levels of serum inflammatory factors (IL-1*β*, MCP-1, and TGF-*β*1) in rats were measured by the corresponding enzyme-linked immunosorbent assay kits. NS model rats were induced by adriamycin (ADR) and then treated with low- (1 g/kg/d), mid- (2 g/kg/d), high-dose (4 g/kg/d) FHD or/and 740Y-P (a PI3K activator). ^*∗∗∗*^*P* < 0.001 compared with the control group; ^##^*P* < 0.01 and ^###^*P* < 0.001 compared with the model group; ^&^*P* < 0.05 and ^&&&^*P* < 0.001 compared with the low-FHD group; and ^∆∆^*P* < 0.01 and ^∆∆∆^*P* < 0.001 compared with the FHD + 740Y-P group.

The therapeutic effect and mechanism of FHD on NS were next validated in NS rats induced by ADR. As depicted in Figure 7(a), NS rats showed evident loss of body weight when compared with control rats (*P* < 0.05). After administration of high-dose FHD, the body weight of NS rats was significantly increased (*P* < 0.05; Figure 7(a)). The increase of kidney weight is an important indicator of kidney tissue damage [26]. In this study, the right kidney index of NS rats markedly increased versus control rats, which was dose-dependently prevented by treating with FHD (*P* < 0.001; Figure 7(b)). Proteinuria is also a critical biomarker for diverse renal diseases. In this work, the 24 h urines of rats after 4-week drug administration were collected and the level of urine protein was determined. As expected, NS rats presented severe proteinuria as evidenced by the remarkable increase in the excretion of urine protein compared with control rats (*P* < 0.001). The administration of FHD markedly reduced the level of urine protein in NS rats (*P* < 0.001; Figure 7(c)). Moreover, inflammatory response in kidney is an important characteristic of NS, and IL-1*β*, MCP-1, and TGF-*β*1 are critical proinflammatory cytokines [27]. ELISA showed that the levels of serum IL-1*β*, MCP-1, and TGF-*β*1 were significantly increased in NS rats, but FHD treatment attenuated the secretion of these inflammatory cytokines (*P* < 0.01; Figures 7(d)–7(f)). In addition, several reports have indicated that PI3K-Akt signaling pathway acts as a pivotal role in kidney diseases [28–30]. Network pharmacological analysis also proved the essential role of PI3K-Akt pathway in FHD treating NS. In the present study, we found that 740Y-P (a PI3K activator) treatment significantly weakened the therapeutic effect of FHD on NS rats as indicated by the decreased body weight and the increased right kidney index, urine protein level, and inflammatory cytokines (*P* < 0.05; Figures 7(a)–7(f)).

#### 3.5.2. Effects of FHD on Renal Function and Histopathological Changes in NS Rats via Affecting PI3K-Akt Signaling Pathway

**Figure 8:**
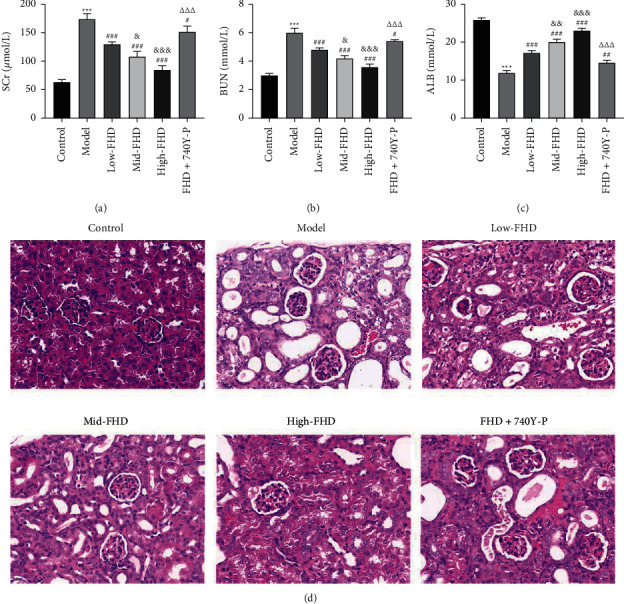
Effects of FHD on renal function and histopathology in NS rats via affecting PI3K-Akt signaling pathway. (a–c) The serum levels of renal function indicators (SCr, BUN, and ALB) were detected using a chemical analyzer. (d) Histopathological changes were measured using the hematoxylin and eosin staining. Scale bar = 20 *μ*m. NS model rats were induced by ADR and then treated with low-, mid-, and high-dose FHD or/and 740Y-P. ^*∗∗∗*^*P* < 0.001 compared with the control group; ^##^*P* < 0.01 and ^###^*P* < 0.001 compared with the model group; ^&^*P* < 0.05, ^&&^*P* < 0.01, and ^&&&^*P* < 0.001 compared with the low-FHD group; and ^∆∆∆^*P* < 0.001 compared with the FHD + 740Y-P group.

The therapeutic effects of FHD on renal function and histopathological changes induced by NS were further evaluated. SCr, BUN, and ALB are three critical indicators widely used to identify kidney function [31]. As shown in Figures 8(a)–8(c), the levels of SCr and BUN were significantly increased in serum of NS rats, and ALB level was decreased in comparison to that in control rats (*P* < 0.001). FHD treatment markedly downregulated the abundance of SCr and BUN and upregulated the ALB level in NS rats in a dose-dependent manner, but the effects of FHD was weakened by 740Y-P addition (*P* < 0.05; Figures 8(a)–8(c)). Additionally, compared with control rats, H&E staining exhibited the serious renal histological damage in NS rats with thickened glomerular basement membrane, hollow renal tubules, and glomerular and tubular atrophy. However, these histopathological indications were mitigated after FHD treatment, in particular high-dose FHD treatment, whereas 740Y-P addition weakened the effect of FHD (Figure 8(d)).

### 3.6. Confirmation of Hub Targets for FHD Treating NS

**Figure 9:**
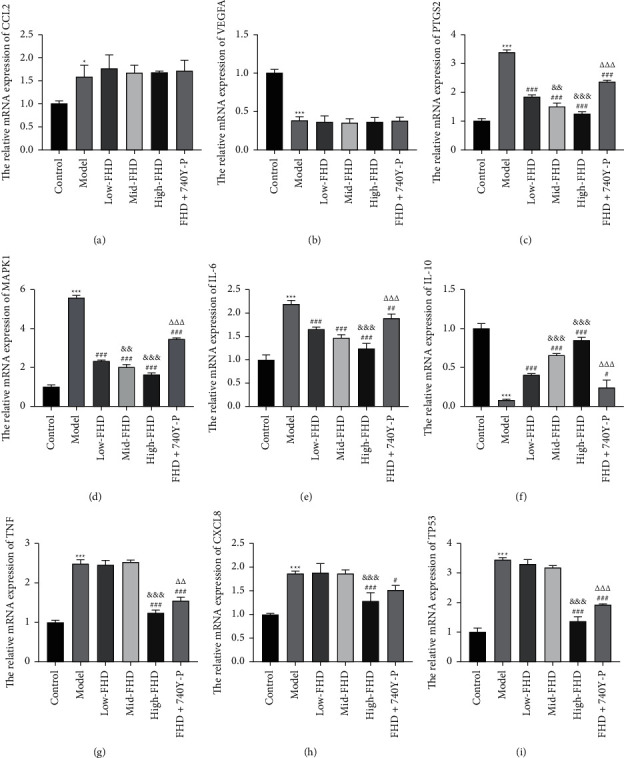
Validation of hub targets for FHD against NS by qRT-PCR. (a–i) The relative mRNA expression of CCL2, VEGFA, PTGS2, MAPK1, IL-6, IL-10, TNF, CXCL8, and TP53. NS model rats were induced by ADR and then treated with low-, mid-, and high-dose FHD or/and 740Y-P. ^*∗*^*P* < 0.05 and ^*∗∗∗*^*P* < 0.001 compared with the control group; ^#^*P* < 0.05, ^##^*P* < 0.01, and ^###^*P* < 0.001 compared with the model group; ^&^*P* < 0.05 and ^&&&^*P* < 0.001 compared with the low-FHD group; *P* < 0.01 and ^∆∆∆^*P* < 0.001 compared with the FHD + 740Y-P group.

**Figure 10:**
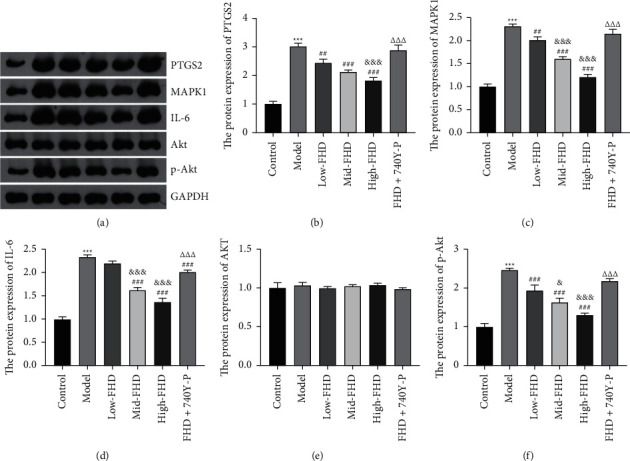
Effects of FHD on the expression of PTGS2, MAPK1, IL-6, and PI3K-Akt pathway in NS rats. (a–f) The relative protein expression of PTGS2, MAPK1, IL-6, Akt, and p-Akt was measured by Western blotting. NS model rats were induced by ADR and then treated low-, mid-, and high-dose FHD or/and 740Y-P. ^*∗∗∗*^*P* < 0.001 compared with the control group; ^##^*P* < 0.01 and ^###^*P* < 0.001 compared with the model group; ^&^*P* < 0.05 and ^&&&^*P* < 0.001 compared with the low-FHD group; and ^∆∆∆^*P* < 0.001 compared with the FHD + 740Y-P group.

Based on the network pharmacological analysis, 9 hug targets (CCL2, IL10, PTGS2, TNF, MAPK1, IL6, CXCL8, TP53, and VEGFA) were collected and validated as the therapeutic targets of FHD on NS in vivo. qRT-PCR showed the increased CCL2 expression and the decreased VEGFA level in NS rats compared with that in control rats (*P* < 0.05), and there were no significant changes after FHD or/and 740Y-P treatment (Figures 9(a) and 9(b)). The expression of PTGS2, MAPK1, and IL-6 was significantly upregulated in NS rats when compared to that in control rats, whereas IL-10 expression was downregulated (*P* < 0.001). FHD treatment dose-dependently rescued the expression levels of PTGS2, MAPK1, IL-6, and IL-10 in NS rats, whereas 740Y-P addition weakened the effect of FHD (*P* < 0.05; Figures 9(c)–9(f)). For the expression of TNF, CXCL8, and TP53, they were dramatically upregulated in NS rats in comparison to that in control rats (*P* < 0.001). High-dose FHD treatment rescued the expression levels of TNF, CXCL8, and TP53 in NS rats, whereas 740Y-P addition weakened the effects of FHD (*P* < 0.001; Figures 9(g)–9(i)). In addition, Western blotting confirmed the results of qRT-PCR on the expression of PTGS2, MAPK1, and IL-6 (*P* < 0.01; Figures 10(a)–10(d)). Meanwhile, the protein level of p-AKT was increased in NS rats compared to that in control rats, which was rescued by FHD administration in a dose-dependent manner (*P* < 0.05). Similarly, 740Y-P treatment weakened the inhibitory effect of FHD on p-AKT expression in NS rats (*P* < 0.001; Figures 10(a), 10(e), and 10(f)).

## 4. Discussion

NS is a highly prevalent kidney disease with the clinal triad of heavy proteinuria, edema, and hypoalbuminemia [2]. Currently, most therapeutics for NS mainly target the NS-related secondary symptoms, which cannot prevent the disease from further developing into renal failure [32]. Therefore, more effective NS treatment drugs and strategies still need to be further investigated. In recent years, TCMs are increasingly becoming the promising therapeutic drugs for kidney diseases for their low side effects and commendable pharmacological activities [33]. FHD is a classical TCMs firstly recorded in Synopsis of the Golden Chamber and widely used in the treatment of edema and dysuria [4]. In this study, based on network pharmacology and functional enrichment analyses, 9 hub targets (CCL2, IL-10, PTGS2, TNF, MAPK1, IL-6, CXCL8, TP53, and VEGFA) and PI3K-Akt pathway were predicted as the potential target mechanisms for FHD treating NS. Furthermore, in vivo experiments validated the therapeutic efficacy of FHD against NS and the potential mechanisms by targeting PI3K-Akt pathway, PTGS2, MAPK1, and IL-6.

Based on network pharmacological analysis, 8 main bioactive compounds (palmatine, *β*-elemene, tetrandrine, fangchinoline, bifendate, calycosin, atractylenolide I, and hinesol) of FHD were predicted to have good affinity with 9 hub targets (CCL2, IL-10, PTGS2, TNF, MAPK1, IL-6, CXCL8, TP53, and VEGFA) for NS treatment. It has been reported that CCL2, IL-10, PTGS2, TNF, IL-6, and CXCL8 were closely bound up with the pathogenic process of NS via regulating immunity and inflammation [34–38]. MAPK1 is a member of mitogen-activated protein kinases that are involved in the regulation of NS progression through activating downstream signals [39]. TP53 and VEGFA are also important molecules that act as important roles in renal microangiogenesis and maintain the functions of kidney [40, 41]. In addition, tetrandrine can reduce the levels of inflammatory factors TNF-*α* and IL-6 and elevate IL-10 level in rats with diabetic nephropathy (DN) [42]. Calycosin also can ameliorate diabetes-induced renal inflammation as indicated by the reduced TNF-*α* level [43]. Fangchinoline exhibits the nephroprotective effects in DN rats via attenuating inflammation and MAPK signaling pathway [44]. Molecular docking in this study further showed that these bioactive compounds of FHD interact with hub targets (CCL2, IL-10, PTGS2, TNF, MAPK1, IL-6, CXCL8, TP53, and VEGFA) with best affinity < −5 kcal/mol. These results indicate that these 9 hub targets might play critical roles in the therapeutic effect of FHD against NS.

Furthermore, GO and KEGG pathway enrichment analyses were applied to further illustrate the mechanisms of FHD in NS treatment. The GO enrichment analysis showed that hub genes mainly function in the regulation of NF-*κ*B transcription factor activity and inflammatory response. NF-*κ*B transcription factor is an important regulator for cellular behaviors and immune activation, the inhibition of which can protect against renal inflammation [45]. These further confirm that the therapeutic efficacy of FHD against NS is closely associated with immunoregulation. In KEGG pathway enrichment analysis, hub targets were mainly enriched in Toll-like receptor, NOD-like receptor, and PI3K-Akt signaling pathways. Accumulating evidence has indicated that PI3K-Akt pathway plays an indispensable role in the regulation of kidney diseases [28–30]. Tu Di et al. demonstrated that the inhibition of PI3K/Akt/mTOR pathway can alleviate renal oxidative stress in membranous nephropathy [28]. Yin et al. revealed that tetrandrine protects against membranous glomerulopathy through targeting PI3K-Akt signaling pathway [29]. Liu and He also elucidated that the suppression of PI3K-Akt pathway can alleviate the inflammatory response, thereby ameliorating focal segmental glomerulosclerosis [30]. Therefore, we considered that PI3K-Akt pathway plays an essential role in FHD treating NS.

To validate the molecular mechanisms predicted by network pharmacology and enrichment analyses, an NS rat model was established by ADR induction. The significantly decreased body weight, the increased kidney index, and the disordered pathophysiology were observed in NS rats, indicating that the establishment of NS rat model was successful. FHD dose-dependently inhibited the decreased body weight and the increased kidney index in NS rats, where 740Y-P (a PI3K activator) weakened the effects of FHD. These results preliminary proved the therapeutic efficacy of FHD against NS and revealed the mechanism involving in PI3K-Akt pathway. Massive proteinuria is a typical clinical syndrome of NS, which was exhibited in NS rats. However, FHD treatment reduced the level of urine protein in NS, where 740Y-P weakened the effect of FHD. SCr, BUN, and ALB are critical assessment indicators for renal function, the levels of which in NS rats were reduced after FHD treatment. In addition, FHD reduced the levels of inflammatory factors (IL-1*β*, MCP-1, and TGF-*β*1) in NS rats. These results indicating that FHD can protect renal function and alleviate inflammation in NS. However, 740Y-P weakened the renoprotective effects of FHD in NS, suggesting that FHD treating NS is involved in the regulation of PI3K-Akt pathway. Furthermore, qRT-PCR and Western blot analyses showed that FHD dose-dependently downregulated the expression of PTGS2, MAPK1, IL-6, and p-Akt in NS. Combined with the results of network pharmacology, these discoveries indicate that FHD exerts the therapeutic effect on NS by targeting PTGS2, MAPK1, IL-6, and PI3K-Akt pathway.

## 5. Conclusion

In summary, network pharmacology predicted that the potential molecular mechanisms of FHD against NS were mainly involved in 9 hub targets and PI3K-Akt signaling pathway. In vivo experiments confirmed the therapeutic effect of FHD on NS and revealed that the underlying mechanisms are closely associated with PI3K-Akt pathway, PTGS2, MAPK1, and IL-6. However, the pharmacological and molecular mechanisms of FHD against NS are needed to be investigated more in depth. This study provides a potential renoprotective drug for NS treatment and offers the important foundation for mechanism investigation.

## Figures and Tables

**Table 1:** The chemical structure of bioactive compounds from FHD.

Synonyms	Molecular formula	2D structure
Palmatine	C_21_H_22_NO_4_^+^	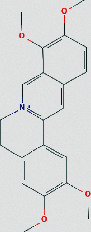
Beta-elemene	C_15_H_24_	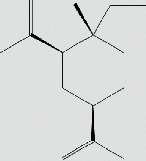
Tetrandrine	C_38_H_42_N_2_O_6_	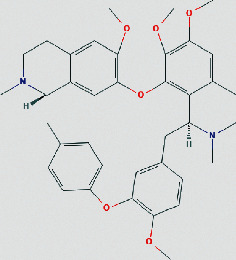
Fangchinoline	C_37_H_40_N_2_O_6_	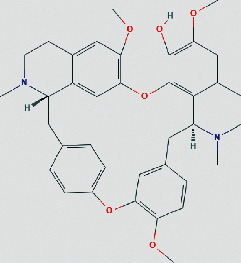
Bifendate	C_20_H_18_O_10_	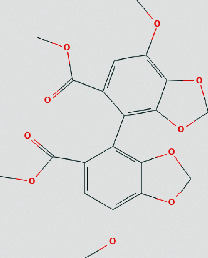
Calycosin	C_16_H_12_O_5_	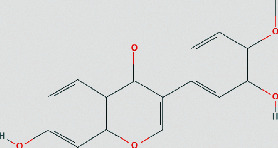
Atractylenolide I	C_15_H_18_O_2_	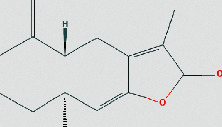
Hinesol	C_15_H_26_O	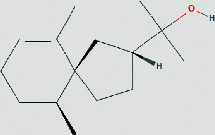

## Data Availability

The raw data used to support the findings of this study are available from the corresponding author upon request.

## Supplementary Materials

Supplementary Materials
*Table S1.* Primer sequences used in qRT-PCR. *Table S2*. A total of 114 bioactive compounds of Fangji Huangqi decoction. *Table S3*. The results of GO enrichment analysis (Top 30). *Table S4*. The results of KEGG enrichment analysis (Top 20). *Table S5*. Molecular docking of bioactive compounds and hub targets.
