# Supplementary material for: Network Pharmacology and In Vivo Experimental Validation to Uncover the Renoprotective Mechanisms of Fangji Huangqi Decoction on Nephrotic Syndrome
Source: Evid Based Complement Alternat Med. 2022 Jun 8;2022:4223729. doi: 10.1155/2022/4223729 (PMC9200505; doi:10.1155/2022/4223729)
Supplement: Supplementary Materials — Table S1. Primer sequences used in qRT-PCR. Table S2. A total of 114 bioactive compounds of Fangji Huangqi decoction. Table S3. The results of GO enrichment analysis (Top 30). Table S4. The results of KEGG enrichment analysis (Top 20). Table S5. Molecular docking of bioactive compounds and hub targets. [file 4223729.f1.zip › 4223729.f1/Revised Table S2.doc]

Table S2: A total of 114 bioactive compounds of Fangji Huangqi decoction

| No. | Molecule names | OB/% | DL |
| --- | --- | --- | --- |
| FJ02 | Hesperetin | 70.31 | 0.27 |
| FJ03 | beta-sitosterol | 36.91 | 0.75 |
| FJ04 | Cissampareine | 9.23 | 0.11 |
| FJ05 | Tetrandrine | 26.64 | 0.10 |
| FJ06 | fangchinoline | - | - |
| FJ07 | Stephanthrine | - | - |
| FJ08 | Beta-Elemene | - | - |
| FJ09 | (+)-2-N-Methyltetrandrine | - | - |
| FJ10 | Palmatine | - | - |
| FJ11 | Stepharine | - | - |
| FJ12 | Cissamine | - | - |
| HQ02 | Jaranol | 50.83 | 0.29 |
| HQ03 | hederagenin | 36.91 | 0.75 |
| HQ05 | isorhamnetin | 49.60 | 0.31 |
| HQ06 | 3,9-di-O-methylnissolin | 53.74 | 0.48 |
| HQ08 | 7-O-methylisomucronulatol | 74.69 | 0.30 |
| HQ10 | (6aR,11aR)-9,10-dimethoxy-6a,11a-dihydro-6H-benzofurano[3,2-c]chromen-3-ol | 64.26 | 0.42 |
| HQ11 | Bifendate | 31.10 | 0.67 |
| HQ12 | formononetin | 69.67 | 0.21 |
| HQ14 | Calycosin | 47.75 | 0.24 |
| HQ15 | kaempferol | 41.88 | 0.24 |
| HQ19 | 1,7-Dihydroxy-3,9-dimethoxy pterocarpene | 39.05 | 0.48 |
| HQ20 | quercetin | 46.43 | 0.28 |
| HQ21 | 7,2',6'-Trihydroxy-5-Methoxyflavanone |  |  |
| HQ22 | Chrysin |  |  |
| HQ23 | Sebacic Acid |  |  |
| HQ24 | Dihydrooroxylin A |  |  |
| BZ06 | 3β-acetoxyatractylone | 54.07 | 0.22 |
| BZ07 | 8β-ethoxy atractylenolide Ⅲ | 35.95 | 0.21 |
| BZ08 | Atractylenolide III | 68.11 | 0.17 |
| BZ09 | Atractylenolide I | 37.37 | 0.15 |
| BZ10 | Jurubine |  |  |
| BZ11 | Hinesol |  |  |
| GC01 | Inermine | 75.18 | 0.54 |
| GC02 | DFV | 32.76 | 0.18 |
| GC04 | Glycyrol | 90.78 | 0.67 |
| GC05 | Jaranol | 50.83 | 0.29 |
| GC06 | Medicarpin | 49.22 | 0.34 |
| GC09 | Lupiwighteone | 51.64 | 0.37 |
| GC10 | 7-Methoxy-2-methyl isoflavone | 42.56 | 0.2 |
| GC11 | formononetin | 69.67 | 0.21 |
| GC12 | Calycosin | 47.75 | 0.24 |
| GC13 | kaempferol | 41.88 | 0.24 |
| GC14 | naringenin | 59.29 | 0.21 |
| GC15 | (2S)-2-[4-hydroxy-3-(3-methylbut-2-enyl)phenyl]-8,8-dimethyl-2,3-dihydropyrano[2,3-f]chromen-4-one | 31.79 | 0.72 |
| GC16 | euchrenone | 30.29 | 0.57 |
| GC17 | glyasperin B | 65.22 | 0.44 |
| GC18 | glyasperin F | 75.84 | 0.54 |
| GC19 | Glyasperin C | 45.56 | 0.4 |
| GC20 | Isotrifoliol | 31.94 | 0.42 |
| GC21 | (E)-1-(2,4-dihydroxyphenyl)-3-(2,2-dimethylchromen-6-yl)prop-2-en-1-one | 39.62 | 0.35 |
| GC22 | kanzonols W | 50.48 | 0.52 |
| GC23 | (2S)-6-(2,4-dihydroxyphenyl)-2-(2-hydroxypropan-2-yl)-4-methoxy-2,3-dihydrofuro[3,2-g]chromen-7-one | 60.25 | 0.63 |
| GC24 | Semilicoisoflavone B | 48.78 | 0.55 |
| GC25 | Glepidotin A | 44.72 | 0.35 |
| GC26 | Glepidotin B | 64.46 | 0.34 |
| GC27 | Phaseolinisoflavan | 32.01 | 0.45 |
| GC28 | Glypallichalcone | 61.6 | 0.19 |
| GC29 | 8-(6-hydroxy-2-benzofuranyl)-2,2-dimethyl-5-chromenol | 58.44 | 0.38 |
| GC30 | Licochalcone B | 76.76 | 0.19 |
| GC31 | licochalcone G | 49.25 | 0.32 |
| GC32 | 3-(2,4-dihydroxyphenyl)-8-(1,1-dimethylprop-2-enyl)-7-hydroxy-5-methoxy-coumarin | 59.62 | 0.43 |
| GC33 | Licoricone | 63.58 | 0.47 |
| GC34 | Gancaonin A | 51.08 | 0.4 |
| GC35 | Gancaonin B | 48.79 | 0.45 |
| GC37 | 3-(3,4-dihydroxyphenyl)-5,7-dihydroxy-8-(3-methylbut-2-enyl)chromone | 66.37 | 0.41 |
| GC38 | 5,7-dihydroxy-3-(4-methoxyphenyl)-8-(3-methylbut-2-enyl)chromone | 30.49 | 0.41 |
| GC39 | 2-(3,4-dihydroxyphenyl)-5,7-dihydroxy-6-(3-methylbut-2-enyl)chromone | 44.15 | 0.41 |
| GC40 | Glycyrin | 52.61 | 0.47 |
| GC41 | Licocoumarone | 33.21 | 0.36 |
| GC42 | Licoisoflavone | 41.61 | 0.42 |
| GC43 | Licoisoflavone B | 38.93 | 0.55 |
| GC44 | licoisoflavanone | 52.47 | 0.54 |
| GC45 | shinpterocarpin | 80.3 | 0.73 |
| GC46 | (E)-3-[3,4-dihydroxy-5-(3-methylbut-2-enyl)phenyl]-1-(2,4-dihydroxyphenyl)prop-2-en-1-one | 46.27 | 0.31 |
| GC47 | liquiritin | 65.69 | 0.74 |
| GC48 | licopyranocoumarin | 80.36 | 0.65 |
| GC50 | Glyzaglabrin | 61.07 | 0.35 |
| GC51 | Glabridin | 53.25 | 0.47 |
| GC53 | Glabrene | 46.27 | 0.44 |
| GC55 | 1,3-dihydroxy-9-methoxy-6-benzofurano[3,2-c]chromenone | 48.14 | 0.43 |
| GC56 | 1,3-dihydroxy-8,9-dimethoxy-6-benzofurano[3,2-c]chromenone | 62.9 | 0.53 |
| GC57 | Eurycarpin A | 43.28 | 0.37 |
| GC60 | Sigmoidin-B | 34.88 | 0.41 |
| GC61 | (2R)-7-hydroxy-2-(4-hydroxyphenyl)chroman-4-one | 71.12 | 0.18 |
| GC62 | (2S)-7-hydroxy-2-(4-hydroxyphenyl)-8-(3-methylbut-2-enyl)chroman-4-one | 36.57 | 0.32 |
| GC63 | Isoglycyrol | 44.7 | 0.84 |
| GC64 | Isolicoflavonol | 45.17 | 0.42 |
| GC65 | HMO | 38.37 | 0.21 |
| GC66 | 1-Methoxyphaseollidin | 69.98 | 0.64 |
| GC67 | Quercetin der. | 46.45 | 0.33 |
| GC68 | 3'-Hydroxy-4'-O-Methylglabridin | 43.71 | 0.57 |
| GC69 | licochalcone a | 40.79 | 0.29 |
| GC70 | 3'-Methoxyglabridin | 46.16 | 0.57 |
| GC71 | 2-[(3R)-8,8-dimethyl-3,4-dihydro-2H-pyrano[6,5-f]chromen-3-yl]-5-methoxyphenol | 36.21 | 0.52 |
| GC72 | Inflacoumarin A | 39.71 | 0.33 |
| GC74 | Kanzonol F | 32.47 | 0.89 |
| GC75 | 6-prenylated eriodictyol | 39.22 | 0.41 |
| GC76 | 7,2',4'-trihydroxy－5-methoxy-3－arylcoumarin | 83.71 | 0.27 |
| GC77 | 7-Acetoxy-2-methylisoflavone | 38.92 | 0.26 |
| GC78 | 8-prenylated eriodictyol | 53.79 | 0.4 |
| GC80 | Vestitol | 74.66 | 0.21 |
| GC81 | Gancaonin G | 60.44 | 0.39 |
| GC82 | Gancaonin H | 50.1 | 0.78 |
| GC83 | Licoagrocarpin | 58.81 | 0.58 |
| GC84 | Glyasperins M | 72.67 | 0.59 |
| GC85 | Glycyrrhiza flavonol A | 41.28 | 0.6 |
| GC86 | Licoagroisoflavone | 57.28 | 0.49 |
| GC88 | Odoratin | 49.95 | 0.3 |
| GC89 | Phaseol | 78.77 | 0.58 |
| GC90 | Xambioona | 54.85 | 0.87 |
| GC91 | dehydroglyasperins C | 53.82 | 0.37 |
| GC92 | quercetin | 46.43 | 0.28 |
| GC93 | isorhamnetin | 49.60 | 0.31 |
