# Supplementary material for: Network Pharmacology and In Vivo Experimental Validation to Uncover the Renoprotective Mechanisms of Fangji Huangqi Decoction on Nephrotic Syndrome
Source: Evid Based Complement Alternat Med. 2022 Jun 8;2022:4223729. doi: 10.1155/2022/4223729 (PMC9200505; doi:10.1155/2022/4223729)
Supplement: Supplementary Materials — Table S1. Primer sequences used in qRT-PCR. Table S2. A total of 114 bioactive compounds of Fangji Huangqi decoction. Table S3. The results of GO enrichment analysis (Top 30). Table S4. The results of KEGG enrichment analysis (Top 20). Table S5. Molecular docking of bioactive compounds and hub targets. [file 4223729.f1.zip › 4223729.f1/Revised Table S3.doc]

Table S3: The results of GO enrichment analysis (Top 30).

| GO term | enrichment | pvalue | count | subgroup |
| --- | --- | --- | --- | --- |
| positive regulation of nitric oxide biosynthetic process | 109.8314 | 6.97E-15 | 9 | Biological process |
| lipopolysaccharide-mediated signaling pathway | 131.1875 | 1.15E-13 | 8 | Biological process |
| positive regulation of transcription from RNA polymerase II promoter | 9.093527 | 2.14E-12 | 17 | Biological process |
| cellular response to lipopolysaccharide | 41.79425 | 2.27E-11 | 9 | Biological process |
| angiogenesis | 23.53139 | 1.71E-10 | 10 | Biological process |
| positive regulation of gene expression | 20.02863 | 7.14E-10 | 10 | Biological process |
| inflammatory response | 15.23021 | 8.86E-10 | 11 | Biological process |
| positive regulation of transcription, DNA-templated | 12.22718 | 9.86E-10 | 12 | Biological process |
| positive regulation of NF-kappaB transcription factor activity | 31.56391 | 3.74E-09 | 8 | Biological process |
| positive regulation of sequence-specific DNA binding transcription factor activity | 34.98333 | 3.36E-08 | 7 | Biological process |
| extracellular space | 8.033036 | 3.17E-13 | 19 | Cellular component |
| extracellular region | 7.074534 | 4.30E-13 | 20 | Cellular component |
| external side of plasma membrane | 13.36854 | 4.46E-04 | 5 | Cellular component |
| cytoplasm | 2.072099 | 5.83E-04 | 19 | Cellular component |
| platelet alpha granule lumen | 31.06364 | 0.003932 | 3 | Cellular component |
| caveola | 26.28462 | 0.005449 | 3 | Cellular component |
| secretory granule | 23.08784 | 0.007009 | 3 | Cellular component |
| extracellular matrix | 7.695946 | 0.013626 | 4 | Cellular component |
| blood microparticle | 11.24013 | 0.027437 | 3 | Cellular component |
| protein complex | 5.529126 | 0.032295 | 4 | Cellular component |
| cytokine activity | 29.97337 | 1.96E-11 | 10 | Molecular function |
| growth factor activity | 26.05093 | 1.43E-08 | 8 | Molecular function |
| enzyme binding | 11.08925 | 2.73E-05 | 7 | Molecular function |
| transcription factor binding | 11.14503 | 1.54E-04 | 6 | Molecular function |
| identical protein binding | 5.634513 | 3.40E-04 | 8 | Molecular function |
| hormone activity | 22.44814 | 6.71E-04 | 4 | Molecular function |
| protease binding | 20.89233 | 8.27E-04 | 4 | Molecular function |
| heparin binding | 13.18828 | 0.003091 | 4 | Molecular function |
| protein binding | 1.501227 | 0.003316 | 25 | Molecular function |
| transcriptional activator activity | 8.941208 | 0.009086 | 4 | Molecular function |
